# Optimized Cardiorespiratory State at Tone Onset Enhances Sensory Responses but Not Associative Learning in Healthy Adults

**DOI:** 10.1111/ejn.70722

**Published:** 2026-10-15

**Authors:** Praghajieeth Raajhen Santhana Gopalan, Miriam S. Nokia

**Affiliations:** ^1^ Department of Psychology and Centre for Interdisciplinary Brain Research University of Jyväskylä Jyväskylä Finland

**Keywords:** ageing, associative learning, cardiac cycle, event‐related potentials, respiration

## Abstract

Previous studies suggest that when a tone‐conditioned stimulus (CS) predicting an airpuff towards the eye is presented at diastole during expiration, both sensory responses to the CS and trace eyeblink conditioning (TEBC) are enhanced. However, not all participants learn and individual variation in the body–brain relationship remains overlooked. We studied whether timing the CS based on an individually optimized bodily state enhances sensory responses and TEBC. We assumed so, as sensory response magnitude reflects attention and stimulus salience: A salient CS should facilitate TEBC. Forty‐one young adults and 19 older adults completed a passive auditory task while electrocardiogram, respiration and electroencephalogram were recorded. The cardiorespiratory state associated with the largest N1–P2 response was identified individually. Optimal states varied across participants and did not differ between age groups. Participants underwent TEBC at their optimal state (Personalized) or at diastole during expiration (Control). As expected, the CS evoked a larger N1–P2 response in the Personalized than the Control group. However, conditioned responding, peak performance, and learning rate, or the proportion of good learners did not differ between groups. Older adults learned more slowly, made fewer conditioned responses, and were less aware of the tone‐airpuff association than young adults. These findings tentatively suggest that enhancing early sensory processing of the CS may not improve TEBC, although the low reliability of the individually determined optimal state limits this conclusion. Future studies should examine whether timing external stimuli to specific bodily states effectively influences behaviour in tasks that rely on rapid sensory processing.

## Graphical abstract

An individually optimized cardiorespiratory state at stimulus onset enhanced brain responses (N1–P2) to a neutral tone compared to those elicited in a separate group of individuals at diastole during expiration. However, optimizing sensory processing of the tone used as a conditioned stimulus did not improve trace eyeblink conditioning.

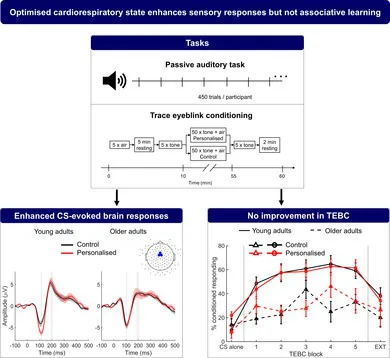

**Abbreviations**
ANOVA: analysis of variance
CR: conditioned response
CS: conditioned stimulus
ECG: electrocardiogram
EEG: electroencephalogram
EMG: electromyography
ERP: event‐related potential
EXP: expiration
DIA: diastole
IBI: inter‐beat interval
ICA: independent component analysis
INS: inspiration
ITI: inter‐trial interval
ITC: inter‐trial coherence
N1: auditory N1 component
P2: auditory P2 component
ROI: region of interest
SD: standard deviation
SEM: standard error of the mean
SYS: systole
TEBC: trace eyeblink conditioning
US: unconditioned stimulus

## 1 Introduction

Brain oscillations shape neural excitability, and the phase of a specific oscillation in relevant brain structures at stimulus onset can influence stimulus processing and learning (Buzsaki 2006; Nokia et al. 2015). Cardiac and respiratory rhythms may have a similar impact on cognition and behaviour. This is thought to arise in part from associations between bodily rhythms and brain oscillations, such as breathing and brain activity in the theta (~4 Hz) range in rodents (Tort et al. 2025). Both oscillatory and aperiodic brain activity seem to be modulated by the phase of the respiratory cycle also in healthy resting adults (Kluger et al. 2023). Further, the connections between body and brain are not limited to respiration but cover various rhythms ranging from slow gastric oscillations to faster repeating events such as the cardiac cycle (for reviews, see Engelen et al. 2023; Parviainen et al. 2022).

In addition to paralleling or possibly governing ongoing endogenous brain activity, bodily rhythms are also linked with stimulus‐evoked brain responses, for example the neural correlates of auditory attention. The N1 component of the auditory event‐related potential (ERP) is larger if selective attention is directed to the stimulus at hand (Hillyard et al. 1973). N1 responses to an acoustic startle stimulus (> 95 dB) are reported to be higher in amplitude at diastole than systole (Schulz et al. 2020). However, the subsequent P2 and P3 components are emphasized at systole rather than diastole (Schulz et al. 2020). Partially consistent with this, auditory P2, but not N1 (see also Li et al. 2024), has been reported to be larger during diastole than systole whether attention was diverted to or away from the stimulus (Mizuhara et al. 2025). P2 was also larger during expiration than inspiration, but only if the participants attempted to ignore the auditory stimulus (Mizuhara et al. 2025). During non‐attended trace eyeblink conditioning (TEBC) in young adults, a tone‐conditioned stimulus (CS) evoked larger N1 amplitudes during systole than diastole (Waselius et al. 2018). In a follow‐up study using the same paradigm, larger N1 amplitudes were elicited at diastole during expiration than at systole during inspiration (Waselius et al. 2022). To summarize, cardiac and respiratory cycle phases seem to affect neural processing of auditory stimuli, as reflected by ERP N1 and/or P2 amplitudes.

Regarding behaviour elicited by auditory stimuli, an extensive line of work from the Schulz group indicates larger (Schulz et al. 2009) and faster (Schulz et al. 2020) eyeblinks to auditory startle stimuli at diastole than systole, consistent with the larger N1 amplitude (Schulz et al. 2020). Further, higher‐amplitude startle eyeblink responses were elicited at ongoing expiration than at other phases of the respiratory cycle both during free breathing and during instructed breathing at 0.25 Hz (Schulz et al. 2016). Interestingly, during TEBC, presenting a tone‐CS followed by an airpuff‐unconditioned stimulus (US) systematically at systole or diastole did not result in a significant difference in learning at the behavioural level, despite the larger CS‐evoked N1 amplitudes at systole than diastole (Waselius et al. 2018). TEBC trials delivered during expiration, rather than inspiration or at random, resulted in more conditioned responses (Waselius et al. 2019). Further, participants trained at diastole during expiration (EXP–DIA) produced more conditioned responses than those trained at systole during inspiration (INS–SYS), consistent with the difference in the CS‐evoked N1 (Waselius et al. 2022). Finally, older adults (> 70 years) trained at EXP–DIA produced more conditioned responses than those trained at INS–SYS, although the proportion of participants who learned TEBC and CS‐evoked N1 amplitudes did not differ between groups (Santhana Gopalan et al. 2024). To summarize, research suggests that presenting an auditory stimulus during diastole rather than systole, and during expiration rather than inspiration, facilitates both reflexive and learned eyeblinks. However, evidence linking these behavioural effects to differences in neural processing of the stimulus, as indexed by the N1 amplitude, remains inconsistent. Here, we study whether maximizing the sensory brain response to the tone‐CS facilitates TEBC in young and older adults. Our approach is based on several previous experimental findings and assumptions: Cardiorespiratory phase at stimulus onset affects N1 amplitude (Waselius et al. 2022). N1 amplitude is thought to reflect the level of attention devoted to said stimulus (Hillyard et al. 1973). Attention paid to a stimulus is positively associated with its salience (Pearce and Hall 1980). Finally, a more salient CS is thought to be more associable and to lead to more efficient classical conditioning (Pearce and Hall 1980). Thus, we assume that an increase in the CS‐evoked N1 amplitude should lead to better TEBC. To test our assumption, we first determine the cardiorespiratory state at which a neutral tone elicits the highest‐amplitude N1–P2 response for each individual participant. Second, participants are divided into two groups for which TEBC trials are delivered either starting at the individually determined optimal cardiorespiratory state (Personalized) or at EXP–DIA (Control), previously deemed beneficial for learning (Santhana Gopalan et al. 2024; Waselius et al. 2022). We expect to see larger N1–P2 responses to the CS and better learning in the Personalized than the Control group. In addition, given the known age‐related alterations in autonomic regulation and neural plasticity (Cabeza et al. 2018; Shaffer and Ginsberg 2017), we expect better learning in young than in older adults (Cheng et al. 2010; Knuttinen et al. 2001).

## 2 Materials and Methods

### 2.1 Participants and Ethics Statement

The study was conducted in compliance with the Declaration of Helsinki, and the protocols were approved by the ethics committee of the University of Jyväskylä prior to the start of the experiment. Participants were recruited based on age and self‐reported health status via university email lists and public advertisements at various events. Exclusion criteria were as follows: severe vision or hearing loss, diagnosed psychiatric, neurological, or neurodegenerative disorders including epilepsy and dementia, a history of head injury, chronic headache or acute migraine, diagnosed cardiac arrhythmia, chronic lung disease, attention deficit disorder or language‐specific disorder, and regular use of medications known to affect the central nervous system or cardiac function (e.g., antidepressants, benzodiazepines, or other psychoactive or heart‐rate–modulating drugs). All participants provided informed consent prior to the experiment. A total of 60 participants were recruited, comprising 41 young adults (13 males, 28 females) aged 20–45 years (mean ± standard deviation: 28 ± 6 years) and 19 older adults (6 males, 13 females) aged 55–80 years (mean ± standard deviation: 70 ± 7 years). After completing the experiment, participants were offered one packet of coffee, tea or candy worth less than 10 euros as a reward. The reward came as a surprise to the participants.

### 2.2 Physiological Measures

Electroencephalography (EEG) was recorded using a high‐density HydroCel Geodesic Sensor Net comprising 128 Ag–AgCl electrodes (Electrical Geodesics Inc.). Electrode impedance was maintained below 50 kΩ for most channels, and signal quality was continuously monitored during data acquisition. EEG signals were acquired with an online Cz reference and band‐pass filtered between 0.16 and 250 Hz. Cardiac activity was measured using three electrocardiogram (ECG) electrodes (Kendall, H92SG), positioned on the right clavicle, the left lower rib cage, and the back of the neck for grounding. Electromyographic (EMG) activity was recorded with two surface electrodes (7000‐K/12; Ambu, Ballerup, Denmark) placed adjacent to each other beneath the right eye over the orbicularis oculi muscle. Respiratory activity was measured with a reusable fabric belt sensor (RESPA00000; Spes Medica, Italy) secured around the lower chest over clothing. All physiological signals were digitized at a sampling rate of 1 kHz using the NeurOne system (Bittium Biosignals Ltd., Finland).

### 2.3 Passive Auditory Task

The entire experiment (Figure 1) was conducted in a dimly lit, sound‐attenuated room at the premises of the Department of Psychology, University of Jyväskylä. Participants were seated comfortably on a padded chair approximately 130 cm from a 24‐in. computer screen (resolution 1920 × 1080, refresh rate 60 Hz). Participants watched a silent nature documentary displayed on the screen while auditory stimuli were played through a roof‐mounted speaker above them. They were instructed that questions about the content of the film would be asked at the end of the session to ensure they paid attention to the film and not the tones. The auditory stimulus was a sinusoidal 880‐Hz, ~65‐dB, 200‐ms tone to avoid pre‐exposure to the 440‐Hz conditioned stimulus before TEBC (see Section 2.5). The passive auditory task was intended to identify each participant's optimal cardiorespiratory state for auditory processing. The tone was delivered ~450 times throughout the 20‐min experiment. The inter‐trial interval (ITI) was pseudo‐randomized at 2–6 s. Following the task, a questionnaire (see Data S1) about the content of the nature documentary was administered.

**FIGURE 1:**
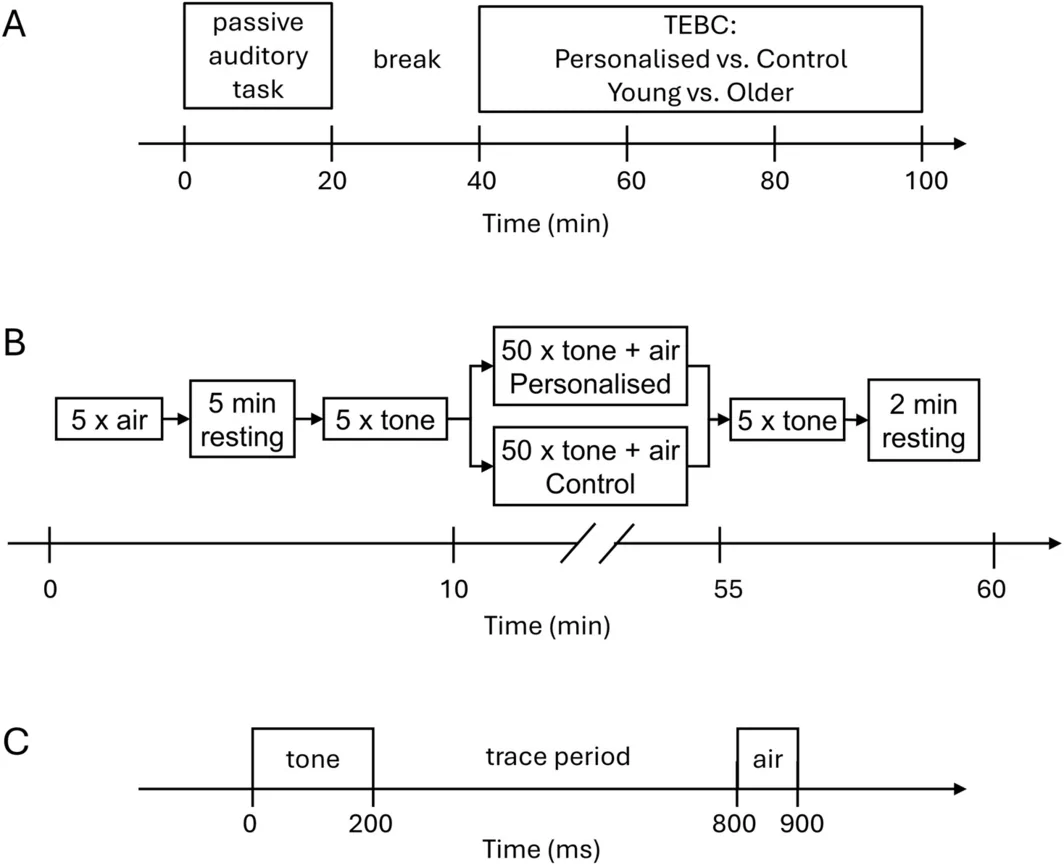
Experimental protocol. (A) Participants first completed a passive auditory task during which a 200‐ms tone was presented 450 times while they watched a silent nature documentary. After a short break, trace eyeblink conditioning (TEBC) was conducted. (B) For the TEBC, participants were divided into two groups: Personalized and Control. The paired trials were timed to start at the individually optimal state in the Personalized group while for everyone in the Control group, the paired trial onset was timed to diastole during expiration. (C) During TEBC paired trials the conditioned stimulus, a 200‐ms tone, was followed by a 600‐ms stimulus‐free trace period and then by a 100‐ms airpuff towards the eye.

### 2.4 Determining Optimal Cardiorespiratory State Based on N1–P2 Amplitude

To continue with the experiment, the experimenter analysed data from the passive auditory task (see below). During this analysis break, all participants regardless of group were allowed to rest, use their mobile phone, read magazines, or use the restroom if needed.

#### 2.4.1 Respiration and Cardiac Cycle Phase at Tone Onset

Respiration and ECG signals recorded during the passive auditory task were used to determine cardiorespiratory state at stimulus onset. Trials from the passive auditory task were sorted into inspiration and expiration bins based on the respiration phase at tone onset. The phase of the respiration signal in radians was derived at each time point using the Hilbert transform implemented in MATLAB. The respiration cycle, ranging from −π to π radians, was divided into two bins: inspiration and expiration. A cutoff at 0 rad was applied, such that negative phase values (−π to 0 rad) were classified as expiration and positive values (0 to π radians) as inspiration. Phase values were rounded to two decimal places to avoid binning errors caused by small numerical differences. Across all participants, there were on average 184 ± 26 trials per participant during inspiration and 247 ± 27 trials per participant during expiration. For quality control, we visually inspected all participants to confirm that the Hilbert‐derived phase trace aligned with the raw respiration waveform, such that the zero‐radian crossing coincided with peak inspiration and −π/π radians corresponded to the end of expiration.

Trials from the passive auditory exposure task were assigned to systole or diastole based on the time elapsed from the most recent R‐peak to the tone onset. R‐peaks were detected from the ECG signal using MATLAB. For each trial, the time difference between the tone onset and the nearest preceding R‐peak was computed. To determine participant‐specific cardiac phase durations, inter‐beat intervals (IBIs) were extracted from the ECG signal, and ECG segments were created around each R‐peak. These segments were averaged to obtain a representative cardiac cycle waveform for each participant. Based on the averaged ECG waveform, the QRS complex and subsequent relaxation phases were identified, and the cardiac cycle was divided into two bins relative to the R‐peak: systole and diastole. Cutoff time points for these phases were defined relative to the R‐peak and adjusted individually for each participant based on their mean IBI, thereby accounting for inter‐individual variability in cardiac cycle duration. Across all participants, there were on average 175 ± 25 trials per participant during systole and 256 ± 26 trials per participant during diastole.

#### 2.4.2 Auditory N1–P2 Amplitude at Different Cardiorespiratory States

EEG data from the passive auditory experiment were preprocessed using EEGLAB (Delorme and Makeig 2004) and FieldTrip (Oostenveld et al. 2011) in MATLAB. Continuous EEG recordings were visually inspected, and noisy channels were interpolated using EEGLAB. Independent component analysis (FastICA) was applied, and components reflecting ocular artefacts (eye blinks and eye movements), line noise, movement artefacts, and cardiac activity were identified and removed using the ICLabel plugin. The cleaned EEG data were imported into FieldTrip for further analysis. Data were band‐pass filtered between 1 and 30 Hz, segmented from −200 to 1000 ms relative to tone onset, and baseline‐corrected using the prestimulus interval. Trials were rejected using an automated amplitude‐based procedure: trials were excluded if any channel exceeded 300 μV or if more than five channels exceeded 175 μV. This resulted in a low rejection rate (4.11% ± 7.05% per participant), with 432 ± 32 trials retained per participant. The number of retained trials ranged from 337 to 450 across participants. The remaining trials were re‐referenced to the common average.

Trials were then sorted into four bins based on the respiration and cardiac cycle phase at tone onset: INS–SYS, INS–DIA, EXP–SYS and EXP–DIA. For each bin, ERPs were calculated by averaging data across all trials in the bin from a fronto‐central region of interest (electrodes 6, 7 and 106). Then, three characteristic points were identified from each ERP waveform: (a) the first positive peak following stimulus onset, (b) the subsequent negative trough (corresponding to the N1 component), and (c) the following positive peak (corresponding to the P2 component). Peak detection was first performed automatically within predefined latency windows and subsequently visually verified to ensure accurate identification of waveform morphology. The first positive peak (a) was detected within 50–120 ms after stimulus onset, the N1 trough (b) within 80–160 ms, and the P2 peak (c) within 150–250 ms. A composite amplitude was calculated as: [(𝑎 − 𝑏) + (𝑐 − 𝑏)] /2 (see Figure 2). By incorporating both the negative deflection and the subsequent positive rebound, this measure reduces sensitivity to single‐point noise and reflects the overall dynamic response of the auditory cortical complex rather than isolated peak amplitude. A comparable method has been previously used to quantify local‐field potential population spike amplitude (see, for example, Nokia et al. 2023). For each participant assigned to the Personalized group, the cardiorespiratory state (1 out of 4) yielding the largest N1–P2 amplitude was determined.

**FIGURE 2:**
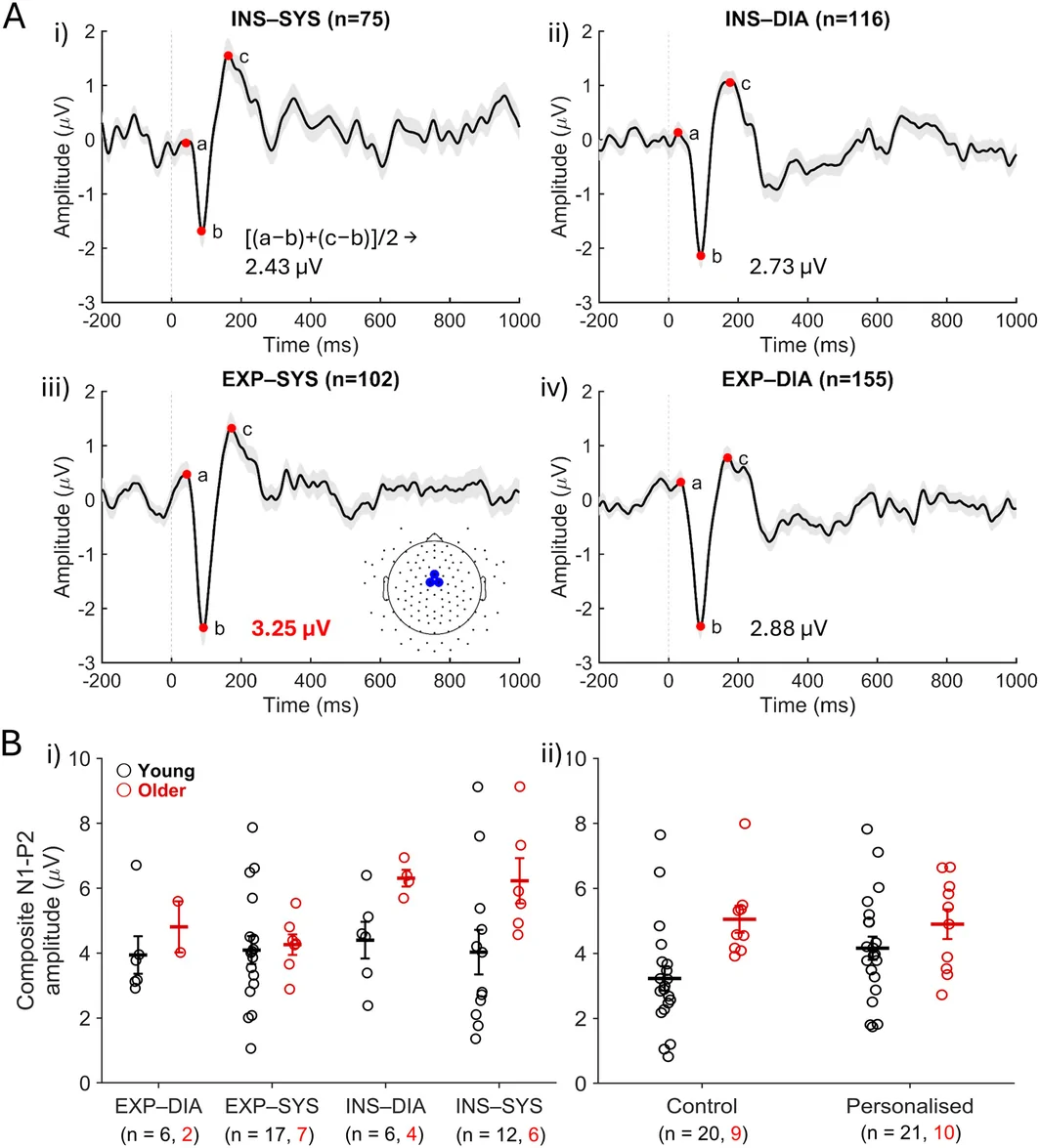
Composite N1–P2 amplitude to the tone during the passive auditory task was used to determine the optimal cardiorespiratory state for TEBC. (A) To assign an optimal TEBC training protocol to each participant in the Personalized group, data from the passive auditory task were processed immediately after the task: Trials (*n*) were divided into four categories or bins based on cardiorespiratory state at tone onset (0 ms): EXP–DIA, EXP–SYS, INS–DIA and INS–SYS. The amplitude of the auditory N1–P2 complex was determined from bin‐average ERPs recorded from a fronto‐central region of interest [blue dots in panel (iii) inset] using a composite peak–trough–peak measure calculated as [(*a* − *b*) + (*c* − *b*)] /2. The optimal state for subsequent TEBC was deemed to be the one at which the N1–P2 complex amplitude was maximal (here, EXP–SYS). Event‐related potentials (ERPs) from a representative young adult participant are illustrated. Red dots on the waveforms indicate the automatically detected timings of peaks a, b and c. Shaded regions represent the standard error of the mean. (Bi) Maximal N1–P2 response amplitude during the passive auditory task for all participants (*n* = 60). Young adults are illustrated with black circles and older adults with red circles. Numbers below the x‐axis indicate the number of participants with maximal responses in that state (EXP‐DIA, EXP–SYS, INS–DIA and INS–SYS). (Bii) Mean N1–P2 response amplitude across all passive auditory task trials for all participants (*n* = 60), illustrated separately for the Control and Personalized groups. Horizontal lines indicate group means, and error bars represent ± SEM. Note that all participants assigned to the Control group were trained at EXP–DIA. SYS = systole, DIA = diastole, EXP = expiration, INS = inspiration.

Note that after the analysis break, all electrode impedances were checked and adjusted to remain below 50 kΩ. TEBC was then initiated.

### 2.5 TEBC

The TEBC paradigm followed the procedure described in our previous study (Santhana Gopalan et al. 2024) (Figure 1C). The participant was instructed to attend the silent nature documentary film displayed on the screen and to ignore disturbing stimuli (i.e., airpuff or tone), as they would later be asked questions related to the content of the film. LabVIEW software (National Instruments, Austin, TX, USA) was used to control stimulus presentation during TEBC. During the TEBC acquisition, respiration and ECG were monitored continuously online. Respiratory state was determined in real time from amplitude‐based features of the respiratory waveform, including respiration rise or fall and peak or trough criteria, while cardiac timing was determined from real‐time ECG R‐peak detection. For each participant, the detection thresholds were set individually by visually inspecting the ECG and respiratory signals in real time to account for individual differences in signal amplitude and morphology. The respiratory and cardiac criteria were combined using predefined logical decision rules. When the required respiratory and cardiac conditions for the target state were simultaneously met, LabVIEW generated a digital output through the data‐acquisition system to initiate the CS. Thus, the real‐time gating procedure was based on amplitude‐based respiratory criteria and R‐peak timing rather than the Hilbert‐transform‐based respiratory phase and offline cardiac‐cycle classification described in Section 2.4.1. The stimuli used and the protocol were the same as in (Santhana Gopalan et al. 2024). Briefly, the experiment started with five US‐alone trials (~2‐bar, 100‐ms airpuff towards eye, 5‐s ITI) followed by a 5‐min period of recording without stimulation. Then, five CS‐alone (tone: 200 ms, 440 Hz, ~66 dB) trials were presented every 20–40 s and participants confirmed that they could hear the tone. During the acquisition phase, 50 CS–US paired trials were presented every 20–40 s, plus the time required for the LabVIEW system to detect the target cardiorespiratory state. During each paired trial, the CS was followed by a 600‐ms stimulus‐free trace interval and then the 100‐ms US. Importantly, in each paired trial, the CS onset was timed to the desired cardiorespiratory state for the participants in the Personalized group and to EXP–DIA for all participants in the Control group. Finally, five CS‐alone trials were presented (extinction). Physiological data were recorded for an additional 2 min while participants continued watching the film. Finally, participants completed a questionnaire assessing attention to the film and awareness of stimulus contingencies (see Data S1). Awareness of the CS–US contingency was assessed using one open‐ended question (not analysed) and seven true/false questions probing knowledge of the temporal order and predictability of the tone and the airpuff. Each correct response to the seven true/false questions was assigned 1 point and each incorrect response 0 points, resulting in an awareness score ranging from 0 to 7, with higher scores indicating greater awareness of the CS–US contingency. The total duration of the TEBC session was approximately 60 min.

To verify the accuracy of CS timing, we retrospectively determined the cardiorespiratory state at CS onset for each of the 50 CS–US paired acquisition trials using the recorded ECG and respiration signals. Each CS onset was classified as inspiration or expiration based on the respiration signal and as systole or diastole based on the ECG signal, resulting in four possible cardiorespiratory states: EXP–DIA, EXP–SYS, INS–DIA and INS–SYS. The observed cardiorespiratory state was then compared with the intended state.

### 2.6 Post‐Experiment Data Analysis

#### 2.6.1 Heart Rate and Respiration Frequency

Heart rate and respiration frequency were calculated offline for each experimental segment using MATLAB (MathWorks, Natick, MA, USA).

#### 2.6.2 Brain Responses

##### 2.6.2.1 N1–P2 Amplitude

EEG data recorded during the TEBC task were preprocessed using the same pipeline as described for the passive auditory task (see above). For TEBC, ERP analyses were restricted to CS–US paired trials. EEG data were segmented into epochs time‐locked to the CS onset (−100 to 500 ms) and averaged to obtain ERPs. Composite N1–P2 amplitudes were computed using the same approach as for the data from the passive auditory task (see above).

##### 2.6.2.2 Induced Power and Inter‐Trial Coherence at the Theta‐Band (5–10 Hz)

To further characterize the neural processes underlying group differences in the composite N1–P2 response during the 50 paired TEBC trials, time–frequency analysis was performed on the EEG data. Analyses focused on a predefined fronto‐central region of interest (signal from electrodes 6, 7 and 106), where the ERP group effect was maximal. ICA‐cleaned EEG data were segmented into epochs from −1000 to 1000 ms relative to tone‐CS onset to reduce edge effects during decomposition. Signals were band‐pass filtered between 1 and 30 Hz, notch filtered at 50 Hz, re‐referenced to the common average, and baseline corrected relative to a −300 to −50 ms prestimulus interval. Single‐trial time–frequency decomposition was computed using wavelets (3 cycles) from 4 to 15 Hz and from −300 to 500 ms relative to tone‐CS onset. Two measures were derived: inter‐trial coherence (ITC), reflecting phase‐locking of responses to the CS onset and induced power, indexing the amplitude of the band‐pass filtered signal and nonphase‐locked activity after removal of the averaged evoked response. For statistical analyses, mean values were extracted from the 5–10 Hz frequency band also called theta‐band (see Herweg et al. 2020), and the 80–300 ms time window following the tone‐CS onset, which corresponds to the timing of the N1 and P2 in the ERP.

### 2.7 Conditioned Eyeblinks

Electromyographic (EMG) activity was recorded from the right orbicularis oculi muscle to quantify conditioned eyeblink responses during TEBC. All preprocessing and analyses were performed in MATLAB using the FieldTrip toolbox. Raw EMG signals were high‐pass filtered at 60 Hz using an 8th‐order zero‐phase (two‐pass) Butterworth filter, rectified, and subsequently low‐pass filtered at 20 Hz using an 8th‐order zero‐phase Butterworth filter to obtain the EMG envelope. The processed signal was segmented into 1500‐ms epochs extending from 500 ms before to 1000 ms after CS onset.

For each trial, the mean (M) and standard deviation (SD) of the rectified EMG amplitude during the 500‐ms pre‐CS baseline period were calculated. An eyeblink was detected when the peak EMG amplitude within a specified time window exceeded a threshold defined as M + 2.5 × SD. Trials were classified as bad and excluded from further analyses if the EMG amplitude exceeded this threshold during the 200‐ms interval immediately preceding CS onset. An adaptively timed conditioned response (CR) was defined as an eyeblink occurring within a 600–800 ms window following CS onset. The first five and last five trials consisted of CS‐only presentations and were excluded from acquisition‐phase analyses. Conditioned responding during learning was quantified using only the CS–US paired trials.

The proportion of trials in which a CR was performed was calculated in five blocks of 10 CS–US trials. The highest percentage of conditioned responding across the final three blocks was used as an index of peak performance. Participants reaching at least 60% CRs across any of the final three acquisition blocks were classified as good learners; the remaining participants were classified as poor learners. Primary conditioning analyses were performed using the full sample (*n* = 60). To maintain consistency with our previous TEBC studies (Santhana Gopalan et al. 2024), the conditioning analyses were also repeated after applying the predefined participant selection criteria used in those studies (participants with > 30% spontaneous blinks during conditioning trials, fewer than five conditioned responses during the conditioning trials, or > 2 spontaneous blinks during the initial CS‐alone trials were excluded), yielding a sample of 45 participants. Learning rate was defined as the number of conditioning trials required to reach the fifth CR during the acquisition phase and was therefore calculated only for participants who reached this criterion.

### 2.8 Statistical Analysis

Statistical analyses were conducted using IBM SPSS Statistics (version 30.0; IBM Corp., Armonk, NY, USA). ERP preprocessing and component extraction were performed in MATLAB using the FieldTrip toolbox. Heart rate and respiration rate were calculated separately for the passive auditory and TEBC sessions, and age group differences were assessed using independent‐samples *t*‐tests.

Learning during TEBC was quantified as the change in the percentage of conditioned responses (CRs) across five successive 10‐trial blocks and analysed using repeated‐measures ANOVA with Block as a within‐subject factor and Age (young vs. older) and Group (personalized vs. control) as between‐subject factors. Violations of sphericity were assessed using Mauchly's test and Greenhouse–Geisser corrections were applied when necessary. Peak performance was defined as the highest CR percentage observed during the final three TEBC blocks and analysed using two‐way between‐subjects ANOVAs with Age and Group as factors. Learning rate, defined as the number of trials required to reach the fifth conditioned response, was analysed using Mann–Whitney *U*‐tests due to non‐normal distribution. To complement the frequentist analyses, Bayesian ANOVAs were performed for the three primary behavioural outcomes (Late CR, peak performance, and learning rate) using JASP (version 0.98.1). Bayes factors for inclusion BF_incl_ were used to quantify evidence for the inclusion of main effects and interactions. Independent‐samples *t*‐tests were used to compare ERP amplitudes between age groups in the passive auditory task. For TEBC, two‐way between‐subjects ANOVAs were conducted with Age and Group as factors to analyse composite N1–P2 amplitudes. Theta band (5–10 Hz) inter‐trial coherence (ITC) and induced power values extracted from the 80–300 ms time window were analysed using two‐way between‐subjects ANOVAs with Age and Group as factors. Pearson correlations and multiple linear regression analyses were used to examine associations between time–frequency measures and composite N1–P2 amplitude, as well as relationships among age, electrophysiological measures, and behavioural learning indices. Chi‐square tests were used to assess distributions of optimal cardiorespiratory state bins and proportions of good learners between groups. Questionnaire performance and awareness scores were analysed using independent‐samples *t*‐tests and two‐way between‐subjects ANOVAs with Age and Group as factors. All tests were two‐tailed with a significance threshold of *p* < 0.05. Effect sizes are reported as eta squared (*η* ^2^) for the ANOVAs examining N1–P2 amplitudes during the passive auditory task, partial eta squared (*ηp* ^2^) for the factorial ANOVAs of TEBC and behavioural outcomes, and *r* for nonparametric tests. Descriptive data are presented as mean ± standard deviation unless otherwise indicated. A sensitivity power analysis was performed using G*Power (version 3.1.9.7) for the primary behavioural analysis. Based on a 2 × 2 factorial ANOVA design, a total sample size of 60 participants, *α* = 0.05, and statistical power of 0.80, the present study was powered to detect effects of Cohen's *f* ≥ 0.368, corresponding to medium‐to‐large effect sizes, and had limited sensitivity to smaller effects.

## 3 Results

### 3.1 Heart Rate and Respiration Rate Did Not Differ Between Young and Older Adults

Data from 60 participants (41 young, 19 older) were included in the analyses of ECG and respiratory data. Heart rate and respiration rate remained within expected ranges during the TEBC session. During the passive auditory task, heart rate averaged at 71.30 ± 10.35 beats/min and respiration rate at 17.76 ± 3.80 breaths/min. During TEBC, heart rate averaged at 67.80 ± 12.73 beats/min and respiration rate at 18.65 ± 2.70 breaths/min. During the passive auditory task, heart rate did not differ significantly between young and older adults, *t*(26.46) = 0.63, *p* = 0.536, nor did respiration rate, *t*(58) = −0.56, *p* = 0.577. A similar pattern was observed during TEBC: Heart rate did not differ significantly between young and older adults, *t*(58) = 1.17, *p* = 0.246, nor did respiration rate, *t*(58) = −0.64, *p* = 0.522. Together, these findings indicate stable cardiorespiratory function across both experimental phases in both age groups.

### 3.2 Maximal N1–P2 Responses During the Passive Auditory Task Were Distributed Across Cardiorespiratory States

Across all participants (*N* = 60), the maximum N1–P2 amplitude occurred during EXP–DIA (*n* = 8; 6 young, 2 older), EXP–SYS (*n* = 24; 17 young, 7 older), INS–DIA (*n* = 10; 6 young, 4 older) and INS–SYS (*n* = 18; 12 young, 6 older), corresponding to 13.3%, 40.0%, 16.7%, and 30.0% of participants, respectively (Figure 2). The distribution of individually determined optimal cardiorespiratory state differed significantly from a uniform distribution, *χ* ^2^(3) = 10.93, *p* = 0.012, with EXP–SYS being the most frequent cardiorespiratory state where maximal auditory responses were elicited. The distribution of optimal physiological states did not differ significantly between young (*n* = 41) and older (*n* = 19) adults, *χ* ^2^(3) = 0.58, *p* = 0.902. To assess the reliability of the individually determined optimal cardiorespiratory state, passive auditory task trials were divided into two independent halves and the optimal state was determined separately for each half. The same optimal state was identified in only 10 of 60 participants (16.7%), whereas the optimal state differed between halves in 50 participants (83.3%; Cohen's *κ* = −0.151). However, the underlying N1–P2 amplitudes showed high split‐half reliability across the four physiological states (EXP–DIA: *r* = 0.862; EXP–SYS: *r* = 0.863; INS–DIA: *r* = 0.885; INS–SYS: *r* = 0.883; all *p* < 0.001).

Across all participants (*N* = 60), the corresponding maximal N1–P2 amplitudes were 4.16 ± 1.33 μV (EXP–DIA, mean ± standard deviation), 4.14 ± 1.54 μV (EXP–SYS), 5.16 ± 1.46 μV (INS–DIA) and 4.76 ± 2.38 μV (INS–SYS). The maximal N1–P2 amplitude was significantly higher in young than older adults, *F*(1,52) = 5.84, *p* = 0.019, *η* ^2^ = 0.090, but did not differ significantly between the four physiological states, *F*(3,52) = 1.48, *p* = 0.231, η^2^ = 0.068. The age × state interaction was not significant, *F*(3,52) = 1.20, *p* = 0.319, η^2^ = 0.055. The mean N1–P2 amplitude across all passive auditory task trials also differed significantly between young and older adults, *F*(1,56) = 8.69, *p* = 0.005, *η* ^2^ = 0.128, with no significant effect of the subsequent TEBC training group (Control vs. Personalized), *F*(1,56) = 0.81, *p* = 0.372, *η* ^2^ = 0.012, or age × group interaction, *F*(1,56) = 1.55, *p* = 0.218, *η* ^2^ = 0.023. The maximum N1–P2 amplitude relative to the mean across the four physiological states was 111.63 ± 10.35% in young adults and 108.06 ± 3.71% in older adults. The relative maximum amplitude did not differ significantly between age groups, *t*(58) = 1.45, *p* = 0.151.

To summarize, auditory brain responses were most often largest at EXP–SYS but the split‐half test suggested low reliability. Brain responses to the neutral tone presented prior to conditioning did not differ significantly between the four cardiorespiratory states within subjects or between the two subsequent TEBC training groups but were significantly larger in amplitude in young than older adults.

### 3.3 Brain Responses to the Tone‐CS During TEBC Were Larger in the Personalized Than the Control Group

Next, we analysed brain responses to the tone‐CS during TEBC. Analyses included EEG data from all participants (*n* = 60) (see Figure 3). Because the personalized TEBC was based on the composite N1–P2 measure in the passive auditory task, we examined whether this same composite metric of the tone‐CS‐elicited response was modulated by age or group during TEBC: There was a significant main effect of Group, *F*(1,56) = 5.54, *p* = 0.022, *ηp* ^2^ = 0.090, with larger amplitude in the Personalized than the Control group. The main effect of Age was not significant, *F*(1,56) = 1.52, *p* = 0.223, *ηp* ^2^ = 0.026, nor was the Age × Group interaction, *F*(1,56) = 0.48, *p* = 0.493, *ηp* ^2^ = 0.008.

**FIGURE 3:**
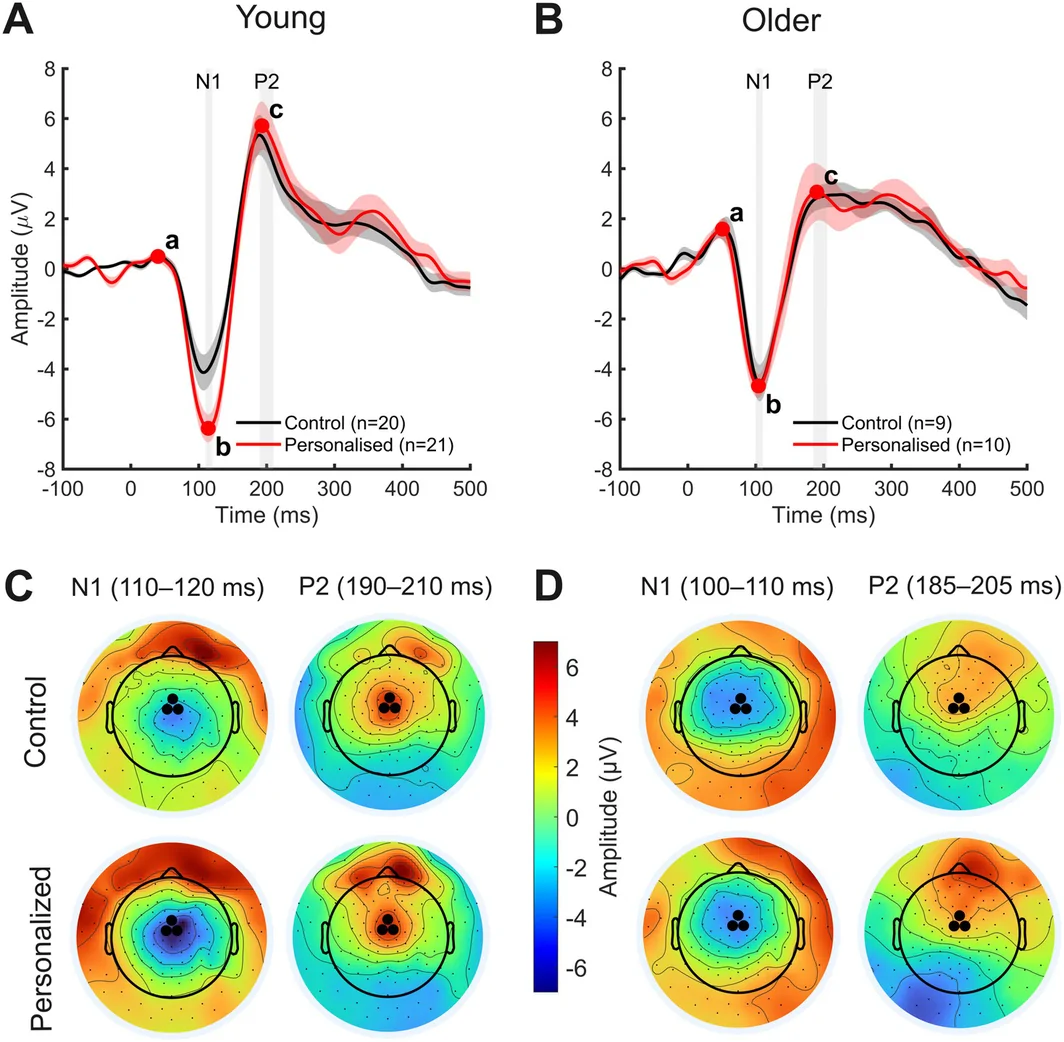
The tone‐CS evoked larger N1–P2 response in the Personalized than the Control group. Grand‐average event‐related potentials (ERPs) to the tone‐CS elicited during paired TEBC trials from a fronto‐central region of interest in young (A) and older (B) adults. Grey shaded regions mark the N1 and P2 time windows and timing of the *a*, *b* and *c* peaks used to determine the composite N1–P2 response amplitude is indicated with red dots. Scalp topographies of mean ERP amplitudes for the N1 and P2 components in young (C) and older (D) adults, shown separately for the Control (top row) and Personalized (bottom row) groups. Topographies are computed as the average voltage within ±5 ms around the group‐level peak latency indicated on top of each plot. Black dots indicate the electrodes included in the region of interest.

We also performed a post hoc validation to determine whether the CS in the 50 CS–US paired acquisition trials was presented in the intended cardiorespiratory state. Across the 3000 CS–US paired trials from all 60 participants, the CS was presented in the intended state in 2528 trials (84.3%). Agreement was 87.0% for EXP–DIA trials, 83.2% for EXP–SYS trials, 79.4% for INS–DIA trials, and 80.0% for INS–SYS trials.

To further characterize the N1–P2 response, we analysed theta band (5–10 Hz) induced power and ITC of the brain responses to the CS. Two‐way ANOVAs (Age × Group) revealed no significant main effect of Group for either ITC (*F*(1,52) = 1.03, *p* = 0.314, *ηp* ^2^ = 0.019) or induced theta power (*F*(1,52) = 1.08, *p* = 0.305, *ηp* ^2^ = 0.020). Likewise, there were no significant Age × Group interactions for ITC (*F*(1,52) = 0.33, *p* = 0.567, *ηp* ^2^ = 0.006) or induced theta power (*F*(1,52) = 0.27, *p* = 0.603, *ηp* ^2^ = 0.005). A multiple regression model including both theta band ITC and induced power as predictors was significant (*F*(2,57) = 35.05, *p* < 0.001), explaining 55% of the variance in the composite N1–P2 amplitude (*R* ^2^ = 0.55). Both ITC (*t* = 7.09, *p* < 0.001) and induced power (*t* = 6.60, *p* < 0.001) independently predicted composite N1–P2 amplitude, indicating that the enhanced ERP response was associated with both stronger trial‐to‐trial phase consistency and increased nonphase‐locked low‐frequency activity. Altogether, aligning the onset of the TEBC trials to the individually selected optimal cardiorespiratory state enhanced processing of the tone‐CS, as reflected by larger composite N1–P2 amplitudes. Furthermore, the enhanced N1–P2 response was independently predicted by both theta‐band ITC and induced theta power.

### 3.4 Young Adults Learned TEBC Better Than Older Adults but There Was no Significant Difference Between the Personalized and the Control Group

Next, we examined the influence of our experimental manipulation and age on learning in the full sample (*n* = 60; Figure 4A,B). There was a significant main effect of Block, *F*(3.41, 191.06) = 5.10, *p* = 0.001, *ηp* ^2^ = 0.084, indicating an increase in conditioned responding across training. The Block × Age × Group interaction, *F*(3.41, 191.06) = 1.64, *p* = 0.175, *ηp* ^2^ = 0.028 was not significant, nor was the Block × Group interaction, *F*(3.41, 191.06) = 0.57, *p* = 0.656, *ηp* ^2^ = 0.01, or the main effect of Group, *F*(1,56) = 0.17, *p* = 0.684, *ηp* ^2^ = 0.003. However, the main effect of Age was significant, *F*(1,56) = 18.87, *p* < 0.001, *ηp* ^2^ = 0.252, indicating less conditioned responding in older than in young adults.

**FIGURE 4:**
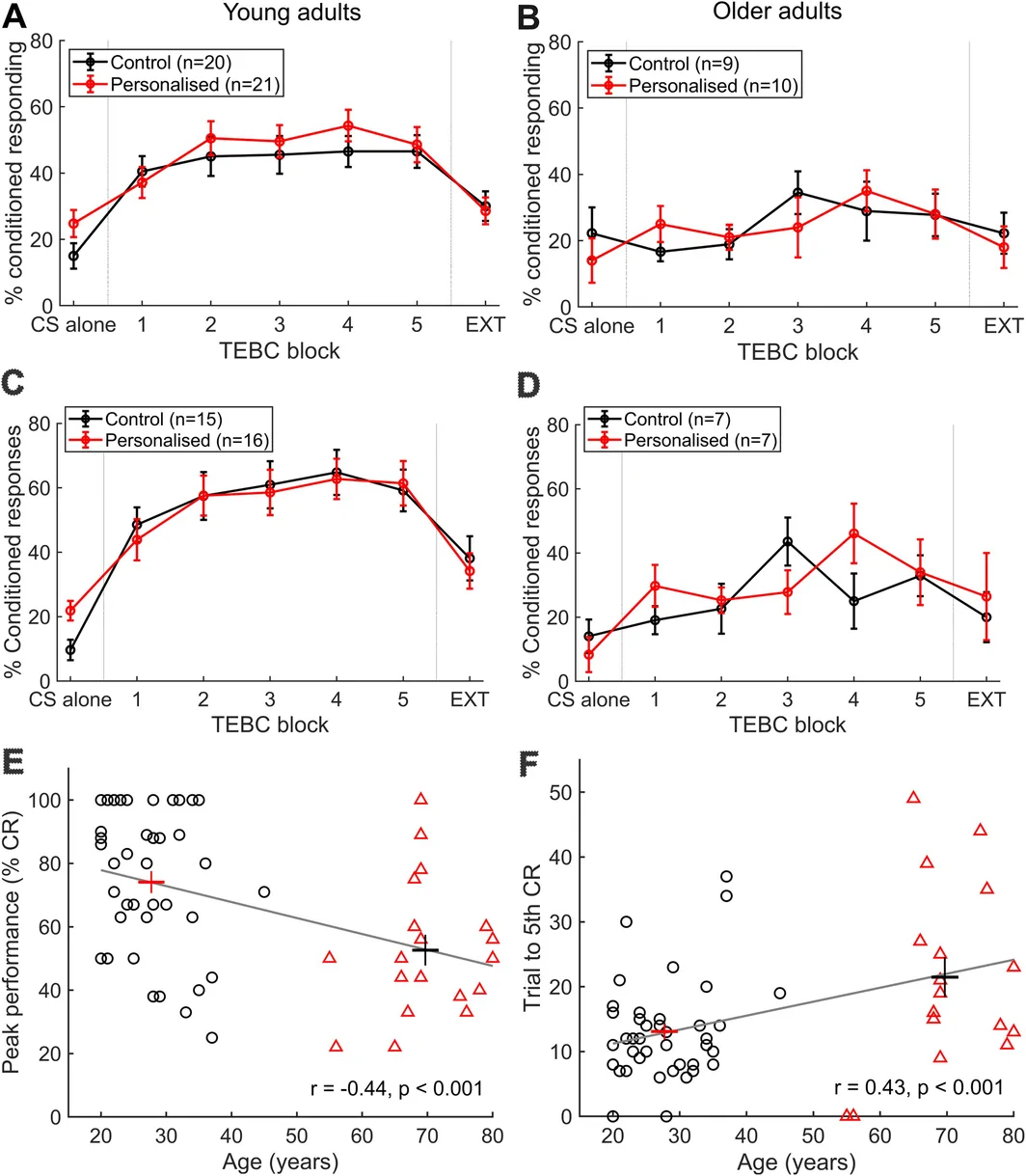
Young adults showed better TEBC learning than older adults, with no significant difference between the Personalized and Control groups. (A) Young adults and (B) older adults: mean percentage of conditioned responses (CRs) across trace eyeblink conditioning (TEBC) for all participants (*n* = 60). The first and final data points represent CS‐alone trials. EXT, extinction. Error bars represent ± SEM. (C) Young adults and (D) older adults: mean percentage of CRs across TEBC for participants with exclusion criteria (*n* = 45; participants with > 30% spontaneous blinks during conditioning trials, fewer than five conditioned responses during the conditioning trials, or > 2 spontaneous blinks during the initial CS‐alone trials were excluded). (E) Relationship between age and peak performance. (F) Relationship between age and learning rate (trials to the 5th CR) across all participants (*n* = 60). Young adults are shown as black circles and older adults as red triangles. Horizontal bars and vertical lines indicate group mean ± SEM (young in red; older in black). Regression lines represent linear fits (*n* = 60). Pearson correlation coefficients (*r*) and associated *p*‐values are displayed within each panel.

Analysing the peak performance, the highest CR% during the last three TEBC blocks (*n* = 60) likewise revealed a significant main effect of Age, *F*(1,56) = 12.07, *p* < 0.001, *ηp* ^2^ = 0.177, no significant effect of Group, *F*(1,56) = 0.23, *p* = 0.635, *ηp* ^2^ = 0.004 and no significant Age × Group interaction, *F*(1,56) = 0.01, *p* = 0.929, *ηp* ^2^ < 0.001 (Figure 4A). Consistent with the significant main effect of Age, a greater proportion of young adults met the good learner criterion (32/41, 78.0%) than older adults (6/19, 31.6%; *χ* ^2^(1) = 12.07, *p* < 0.001). In the full sample, 38/60 participants (63.3%) were classified as good learners. The proportion of good learners did not significantly differ between the Personalized (22/31, 71.0%) and Control groups (16/29, 55.2%; *χ* ^2^(1) = 1.61, *p* = 0.205).

A Mann–Whitney *U*‐test (*n* = 56, excluding participants who did not reach five conditioned responses) indicated that older adults required significantly more trials to reach the 5th CR than young adults (*U* = 132, *Z* = −3.560, *p* < 0.001, *r* = 0.48). No significant difference in learning rate was observed between the Personalized and Control groups (*U* = 326.500, *Z* = −1.067, *p* = 0.286).

To maintain consistency with our previous TEBC studies (Santhana Gopalan et al. 2024), we also repeated the conditioning analyses after applying the predefined participant selection criteria used in those studies (*n* = 45; Figure 4C,D). The overall pattern of results remained unchanged. Conditioned responding across TEBC blocks continued to show significant effects of Block (*F*(3.55,145.70) = 5.09, *p* = 0.001, *ηp* ^2^ = 0.110) and Age (*F*(1,41) = 18.05, *p* < 0.001, *ηp* ^2^ = 0.306), whereas neither the main effect of Group (*F*(1,41) = 0.04, *p* = 0.839, *ηp* ^2^ = 0.001) nor the Age × Group interaction (*F*(1,41) = 0.18, *p* = 0.678, *ηp* ^2^ = 0.004) was significant. Similarly, Late CR (*F*(1,41) = 14.20, *p* < 0.001, *ηp* ^2^ = 0.257) and peak performance (*F*(1,41) = 12.00, *p* = 0.001, *ηp* ^2^ = 0.226) remained significantly lower in older adults, with no significant effects of Group or Age × Group interaction (all *p* ≥ 0.737). Similarly, learning rate (*n* = 44) was analysed using Mann–Whitney *U*‐tests, consistent with the primary analysis. Older adults required more trials to reach the fifth conditioned response than young adults (*U* = 64.000, *Z* = −3.686, *p* < 0.001, *r* = 0.56), whereas learning rate did not differ between the Control and Personalized groups (*U* = 232, *Z* = −0.235, *p* = 0.814, *r* = 0.04).

To further evaluate the nonsignificant behavioural findings, Bayesian ANOVAs were performed for the three primary behavioural outcomes. The analyses provided strong to very strong evidence for a main effect of Age (BF_incl_ = 26.03–131.30). For the two primary conditioning outcomes (Late CR and peak performance; *n* = 60), there was moderate evidence against including a Group effect (BF_incl_ = 0.25–0.27) and an Age × Group interaction (BF_incl_ = 0.32–0.33). For learning rate (*n* = 56), the evidence for a Group effect (BF_incl_ = 1.59) and an Age × Group interaction (BF_incl_ = 1.39) was inconclusive.

To further examine relationships between brain responses to the CS, age and learning, correlations were calculated (*n* = 60). Age was negatively correlated with peak performance (*n* = 60; *r* = −0.44, *p* < 0.001; see Figure 4E) and positively correlated with learning rate (*n* = 56; *r* = 0.43, *p* < 0.001; see Figure 4F). Age was not significantly correlated with composite N1–P2 amplitude (*n* = 60; *r* = −0.15, *p* = 0.249) or induced theta band power (*n* = 60; *r* = −0.13, *p* = 0.307), but older age was associated with lower ITC (*n* = 60; *r* = −0.32, *p* = 0.012). No significant correlations were observed between the N1–P2 amplitude, ITC or induced power at the theta band and any of the measures of conditioned responding (*r* = −0.229 to 0.181, all *p* ≥ 0.089).

In sum, our results indicate that the enhancement of brain responses to the CS during TEBC in the Personalized group compared to the Control group did not translate into improved associative learning. However, young adults learned TEBC better than older adults.

### 3.5 Young Adults Indicated Higher Awareness of the CS–US Contingency Than Older Adults

Finally, we analysed questionnaire responses to assess participants' attention to the silent nature films during the passive auditory task and TEBC. The mean number of correct answers (max. five) based on the film shown during the passive auditory task did not differ between young (4.10 ± 0.77) and older adults (4.33 ± 0.59), *t*(57) = 1.16, *p* = 0.252, or between Personalized (4.29 ± 0.59) and Control groups (4.04 ± 0.84), *t*(57) = 1.36, *p* = 0.179. Regarding the film shown during TEBC, older adults (4.50 ± 0.62) gave more correct answers than young adults (4.05 ± 0.63), *t*(57) = 2.55, *p* = 0.014, with no difference between Personalized (4.20 ± 0.66) and Control group (4.17 ± 0.66), *t*(57) = 0.16, *p* = 0.873.

The mean awareness score was 5.28 ± 0.96 in the Control group and 4.90 ± 1.27 in the Personalized group. There was no significant Age × Group interaction, *F*(1,56) = 1.73, *p* = 0.193, *ηp* ^2^ = 0.030, no significant main effect of Group, *F*(1,56) = 2.84, *p* = 0.098, *ηp* ^2^ = 0.048, but a significant main effect of Age, *F*(1,56) = 4.43, *p* = 0.040, *ηp* ^2^ = 0.073. Young adults scored higher (5.29 ± 0.90) in the questions regarding awareness of the CS–US association than older adults (4.63 ± 1.46).

To summarize, questionnaire results were in line with conditioned responding: Older adults answered more questions correctly regarding the film content but showed lower awareness of the CS–US contingency than young adults.

## 4 Discussion

We studied whether aligning TEBC trials to each participant's optimal cardiorespiratory state would improve learning beyond the EXP–DIA procedure used previously (Santhana Gopalan et al. 2024; Waselius et al. 2022). The cardiorespiratory state associated with the largest N1–P2 response during a passive auditory task varied across participants and did not differ between age groups. Personalized TEBC enhanced the CS‐evoked N1–P2 response but did not improve conditioned responding relative to the Control group trained at EXP–DIA. Older adults learned more slowly and produced fewer conditioned responses than young adults. Finally, at the end of TEBC, young adults were more aware of the CS–US contingency than older adults. Together, these findings provide tentative evidence that enhancing early auditory neural responses to the tone‐CS was not sufficient to improve TEBC. This interpretation should be treated cautiously because the individually determined optimal cardiorespiratory state showed low split‐half reliability.

We found marked interindividual variability in the cardiorespiratory state associated with the largest auditory N1–P2 response elicited during passive listening in young and older adults. When comparing the N1–P2 amplitudes elicited by the neutral tone across the four pre‐determined states within subjects, we detected no statistically significant differences. This indicates that no single cardiorespiratory state consistently maximized auditory responses at the group level. Looking at each participant's data, EXP–SYS was the most frequently identified optimal state, but optimal states varied substantially across participants and even within subjects (low split‐half reliability). That is, determining an optimal state was rather uncertain. However, the underlying N1–P2 amplitudes showed high split‐half reliability, suggesting that the ERP measurements themselves were robust. Thus, the N1–P2 responses were reliable, whereas the specific cardiorespiratory state associated with the largest response varied between trial halves. These observations are somewhat in line with earlier reports: Previous studies have shown that cardiac and respiratory rhythms can modulate neural activity and sensory processing, but the ‘effective state’ seems to depend on the task and measurement used: for example, larger auditory P2 amplitudes have been observed during diastole and expiration (Mizuhara et al. 2025), while visual awareness and ERP responses are enhanced during diastole and inhalation compared to systole and exhalation (Leupin and Britz 2024). Future studies should examine whether a generally optimal physiological state for eliciting N1–P2 can be identified reliably across separate recording sessions and when using different stimuli.

In our current study, we were able to enhance sensory brain responses to the CS yet the alignment of TEBC trials to an individually optimized cardiorespiratory state had no effect on awareness of the CS–US contingency or on learned behaviour. The difference in the N1–P2 responses is in line with previous reports (P2: Mizuhara et al. 2025; N1: Waselius et al. 2018, 2022). The lack of effect on behaviour or awareness is not too surprising taking into account our own prior studies indicating that sensory processing of the CS or awareness of the CS–US contingency do not directly translate into conditioned behaviour (Santhana Gopalan et al. 2024; Waselius et al. 2018) although the two can be associated (Waselius et al. 2022). That is, the brain responses thought to reflect attention or stimulus salience do not straightforwardly connect to or predict classical conditioning. This is in contrast to what we reasoned based on the assumed links from N1 amplitude to attention (Hillyard et al. 1973), from attention to CS salience and from CS salience to classical conditioning (Pearce and Hall 1980). Successful learning likely depends not only on sensory processing of the CS but also on distributed neural networks involved in associative learning: these probably include the cerebellum that controls the adaptive execution and timing of the conditioned eyeblink responses (Thompson and Steinmetz 2009) and forebrain circuits that maintain the memory trace of the CS and tie it with the US across the stimulus‐free trace period (Cheng et al. 2008; Weiss and Disterhoft 2011). In the future, it will be interesting to probe the determinants of successful TEBC further.

Some limitations of our current study should be considered. First, the individually determined optimal cardiorespiratory state showed low split‐half reliability. Although the underlying N1–P2 amplitudes showed high split‐half reliability, the same optimal cardiorespiratory state was identified in only 10 of 60 participants (16.7%). Thus, determining a specific individual optimal state was rather uncertain, which limits the strength of conclusions regarding personalized physiological‐state targeting. Second, the sample size limits the sensitivity of the study to detect small effects. The present design was powered to detect medium‐to‐large effects, and smaller effects may therefore have remained undetected. Third, future studies could improve the sensitivity of TEBC by using more acquisition trials or training to a predefined learning criterion. In addition, examining neural responses beyond the early sensory N1–P2 response, and testing whether timing the US rather than the CS to a specific physiological state influences conditioning may provide further insight into how cardiorespiratory state affects learning.

In summary, personalized timing of the tone‐CS based on cardiorespiratory state enhanced the early neural response but did not improve TEBC beyond the EXP–DIA control condition. That is, enhancing early sensory responses alone is not sufficient to enhance associative learning, which likely depends on additional cerebellar and forebrain processing across training. More broadly, our results highlight the importance of considering both interindividual physiological variability and the functional relevance of selected neural markers when developing practical interventions based on the brain–body connection. Future studies should test whether personalized stimulation based on cardiorespiratory state is more effective in tasks that depend directly on rapid sensory processing.

## Author Contributions

**Praghajieeth Raajhen Santhana Gopalan:** conceptualization, methodology, data curation, investigation, formal analysis, funding acquisition, visualization, project administration, resources, writing – original draft, writing – review and editing. **Miriam S. Nokia:** conceptualization, methodology, supervision, resources, writing – original draft, writing – review and editing.

## Funding

P.R.S.G. was supported by the Faculty of Education and Psychology, University of Jyväskylä, and by the Ella and Georg Ehrnrooth Foundation. This study was also supported by the Academy of Finland (grant no. 355392 to M.S.N.).

## Conflicts of Interest

The authors declare no conflicts of interest.

## Supporting information


**Data S1:** Supporting Information.

## Data Availability

The data that support the findings of the current article are available from the corresponding author upon reasonable request.
