# Supplementary material for: Optimized Cardiorespiratory State at Tone Onset Enhances Sensory Responses but Not Associative Learning in Healthy Adults
Source: Eur J Neurosci. 2026 Oct 15;64(8):e70722. doi: 10.1111/ejn.70722 (PMC13671242; doi:10.1111/ejn.70722)
Supplement: Supplementary file 1 [file EJN-64-0-s001.docx]

Supplementary Material

Optimised cardiorespiratory state at tone onset enhances sensory responses but not associative learning in healthy adults

Running title: Cardiorespiratory rhythm and learning

Praghajieeth Raajhen Santhana Gopalan* and Miriam S. Nokia

Department of Psychology and Centre for Interdisciplinary Brain Research, University of Jyväskylä, Jyväskylä, Finland

*Corresponding author: Praghajieeth Raajhen Santhana Gopalan, [praghajieeth.santhana@jyu.fi](mailto:praghajieeth.santhana@jyu.fi)

Department of Psychology, PO Box 35, FI-40014, University of Jyväskylä, Finland

# Passive auditory task-related questionnaires

The researcher fills in: ID__________ Date__________ Time_________

Please fill in the missing information and answer the following questions.

Age:

Sex:

Handedness:

1. What equipment did they use to walk in the show?
2. Were there any light effects in the film?
3. Name two–three animals that appeared in the film?
4. Did the fishermen catch anything?
5. What large bird appeared at the beginning of the film?

# TEBC-related questionnaires

The researcher fills in: ID__________ Date__________ Time_________

Please answer the following questions.

1. What equipment did the man in the film use for travelling in the snow?
2. Were there some light-phenomena in the film?
3. Mention two–three animals you saw in the film?
4. Did the mushroom pickers find any mushrooms?
5. What bird appeared at the beginning of the second film?
6. How was the airpuff presented during the experiment timed / when did it occur?
7. I believe that the air puff occurred right *before* the beep.	True / False
8. I believe that the air puff occurred right *after* the beep.	True / False
9. I believe that the tone occurred right *before* the air puff.	True / False
10. I believe that the tone occurred right *after* the air puff.	True / False
11. I believe that the tone and the air puff always occurred

very close to each other in time.	True / False

12. I believe that the tone and the air puff occurred close to each other

in time only occasionally.	True / False

13. I believe that the tone predicted the air puff.	True / False
